# Comparative Transduction Profiling of Four Intravenously Delivered AAV Capsids in the Neonatal Murine Nervous System

**DOI:** 10.3390/biomedicines14071426

**Published:** 2026-06-24

**Authors:** Haitong Gao, Tonghui Xu

**Affiliations:** 1 Laboratory Animal Center, Fudan University, Shanghai 200433, China; 2 Laboratory Animal Resource Center, Fudan University, Shanghai 200433, China; 3 School of Life Sciences, Fudan University, Shanghai 200438, China

**Keywords:** AAV capsid, neural transduction, gene delivery

## Abstract

**Background:** Selecting the most efficient and specific adeno-associated virus (AAV) capsids for gene delivery to the nervous system via minimally invasive routes is critical to gene therapy advancement. While AAV9, rAAV2-retro, AAV-PHP.eB, and AAV-MacpnS1 have demonstrated significant central nervous system (CNS) transduction ability after systemic delivery, their tropism, efficiency, and safety profiles in a developmentally relevant model have yet to be systematically compared. This study comparatively evaluated four capsids after intravenous administration in neonatal C57BL/6 mice. **Methods:** Transgene expression was quantitatively assessed across multiple CNS regions, as well as in the heart and liver. Associated biochemical indicators of hepatic stress were also evaluated. **Results:** The resulting transduction profiles were distinct and capsid-specific. Both AAV9 and AAV-MacpnS1 induced widespread CNS transduction and robust peripheral organ expression. However, AAV-MacpnS1-neuronal tropism in the thalamus was superior, and it was also associated with the most prominent biochemical indicators of hepatic stress. In contrast, rAAV2-retro was remarkably specific to the medulla and spinal motor neurons, demonstrating a valuable safety profile. AAV-PHP.eB achieved broad cellular transduction in the spinal cord, but it was the least specific towards cholinergic motor neurons. Furthermore, transduction in DRG neurons using AAV9 and AAV-MacpnS1 was efficient, but that using rAAV2-retro or AAV-PHP.eB was not. **Conclusions:** These findings provide an “atlas-like” comparative framework that clearly outlines the strengths and limitations of each vector. They also offer valuable guidance on selecting the most suitable AAV capsid for fundamental neuroscience applications and for developing targeted gene therapies, particularly for neurodevelopmental and motor neuron disorders, where intravenous administration in the early stages of life is a promising strategy.

## 1. Introduction

The adeno-associated virus (AAV), which is a member of the Parvoviridae family, was first discovered in 1965 as a contaminant of adenovirus preparations [1]. Because it is naturally replication-deficient and non-pathogenic in humans, it requires co-infection with a helper virus for productive replication. This characteristic forms the basis for its development as a safe viral vector for gene therapy [2]. Thanks to its favorable safety profile, sustained transgene expression, and low immunogenicity, the AAV is a leading platform for gene therapy delivery [3]. With a packaging capacity of approximately 4.7 kb, the AAV can carry most therapeutic genes. The scope for its application has widened through capsid engineering and promoter optimization [4]. To date, hundreds of AAV variants, including 13 naturally occurring capsids, have been identified. These capsids exhibit distinct tissue tropisms through their recognition of specific cellular receptors, enabling the development of targeted gene delivery strategies [5,6,7]. For instance, both AAV8 and AAV9 are highly effective mediators of transduction in the liver [5,6]. Additionally, AAV9 also exhibits strong tropism for the skeletal muscle, the heart, and the pancreas [5]. In contrast, AAV1’s expression ability is most effective in the liver, closely followed by its efficiency in the heart and then in the skeletal muscle. AAV6 is particularly efficient in cardiac transduction [7]. The clinical translation of the AAV is underscored by the fact that several therapies using it have been approved by the FDA. AAV2-mediated RPE65 gene therapy (voretigene neparvovec) for Leber congenital amaurosis was approved in 2017, marking the first gene therapy for a monogenic disease in the United States [8]. Promising results have also been reported for AAV5-based therapy (ATSN-101) in patients with type 1 Leber congenital amaurosis caused by GUCY2D mutations [9]. In 2019, the AAV9-based drug onasemnogene abeparvovec (Zolgensma^®^) was approved for the treatment of spinal muscular atrophy [10]. Despite these successes, AAV vectors cannot yet be applied broadly due to challenges, including pre-existing humoral immunity to the capsid, suboptimal transduction efficiency in certain tissues, insufficient cell-type specificity, and dose-dependent toxicity. These challenges limit the use of AAV vectors in specific therapeutic contexts.

The efficient delivery of therapeutic agents to the central nervous system (CNS) remains a fundamental challenge in gene therapy for neurological disorders, largely due to the selective permeability of the blood–brain barrier (BBB). To overcome this challenge, AAV delivery strategies have been broadly categorized into local and systemic administration routes. Local injection approaches, such as intraparenchymal, intracerebroventricular (ICV), and intraspinal delivery, allow for the direct deposition of vectors into or adjacent to the CNS, enabling high local transduction of specific neuronal populations. However, these invasive procedures carry inherent risks, including surgical trauma, hemorrhage, and potential neural damage. They also often fail to achieve widespread or whole-brain transgene distribution. This failure limits their utility for disorders with diffuse pathology [11]. On the other hand, systemic peripheral routes, primarily intravenous (IV) and intramuscular (IM) injections, offer minimally invasive alternatives with the potential for widespread biodistribution in the body. In particular, an IV injection is technically simple and feasible, avoids the morbidity associated with neurosurgery, and shows promise in targeting widespread neuronal and glial populations throughout the central and peripheral nervous systems (CNS and PNS) [12]. Nevertheless, systemic delivery must contend with several biological barriers: the BBB’s significant restriction of viral entry into the CNS; substantial off-target transduction and possible toxicity to the liver caused by intense AAV-related hepatic tropism linked to many capsids; and natural AAV capsids’ habitual failure to be inherently tissue specificity for precise cellular targeting within the nervous system [11,13]. Therefore, while AAV delivery through IV injection is an appealing strategy due to its minimal invasiveness and potential for widespread transduction in the CNS, its application in a clinical setting would require finding a careful balance between achieving adequate therapeutic expression in the nervous system and minimizing off-target effects and systemic toxicity. This balance is particularly crucial when targeting the developing nervous system. The neonatal period offers a unique therapeutic opportunity, as the BBB at this time is more permeable than in adults. Acting on this opportunity could enhance CNS transduction following systemic delivery. Therefore, conducting a systematic comparison in neonates is not only methodologically relevant but also directly informs early-intervention strategies for neurodevelopmental disorders.

The remarkable diversity of AAV capsids—which is the result of variations in capsid protein structure—underlies their distinct tissue tropisms and transduction efficiencies. Recent advances in capsid engineering have expanded this repertoire further, generating novel variants with enhanced properties for targeting the nervous system. Crucially, several of these next-generation AAVs have demonstrated a shared, valuable characteristic: the ability to cause substantial transduction in the CNS following minimally invasive IV delivery, thereby circumventing the need for direct intracranial surgery. Of these next-generation AAVs, four capsids/variants stand out due to their particularly strong affinity for the CNS via systemic administration and are, thus, the focus of this comparative study. AAV9 is a naturally occurring capsid that has long been used for systemic gene delivery. It was initially isolated from human tissues and was later shown by Foust et al. to be able to cross the BBB efficiently, thereby enabling robust transduction of neurons and astrocytes throughout the CNS after IV injection [12,14]. rAAV2-retro, which is engineered through in vivo-directed evolution, has a unique ability to carry out robust retrograde transport [15]. Upon IM or IV injection, this property enables it to efficiently infect projection neurons, including lower motor neurons in the brainstem and spinal cord. This makes it a powerful tool for circuit mapping and motor neuron disease therapy [16]. AAV-PHP.eB, which is a derivative of AAV9 developed by the Gradinaru lab, penetrates the BBB more effectively in specific murine models. This facilitates widespread transduction in the CNS at relatively low viral doses and minimizes peripheral off-targeting [17]. It is efficient therapeutically, as demonstrated in the intrathecal delivery for amyotrophic lateral sclerosis (ALS) [18]. More recently, the engineered variant AAV-MacpnS1 has been explored. It had shown exceptional transduction in the peripheral ganglia (e.g., the dorsal root and nodose ganglia) and the CNS of adult mice following systemic administration. This highlights its capability for dual-compartment targeting [19].

Numerous pairwise comparisons of the distinct properties of the four capsids introduced above have been undertaken, collectively underscoring the necessity and value of a systematic evaluation. These comparisons have shown that AAV9 and AAVrh10 are comparable in their transduction ability in the CNS of neonatal mice. However, AAVrh10 provokes superior expression in the medulla and spinal cord at low doses [20]. Similarly, IV-injected AAV-PHP.eB triggers higher transduction in the CNS of C57BL/6J mice than IV-injected AAV9, with markedly reduced hepatic off-target effects [21]. In the dorsal root ganglia (DRG), exosome-associated AAV9 penetrates the BBB at a higher rate and improves neuronal transduction more effectively than conventional AAV9 [22]. Capsid performance in specific regions, such as the dorsal raphe and cochlea, has seen further scrutiny, yielding valuable results [23,24]. While these pairwise comparisons provide valuable insights, they were conducted under varying experimental conditions, including different routes of administration, different mouse strains, and different dosage regimens, which makes direct cross-study comparisons challenging. Therefore, a direct, head-to-head comparison of these four potent, CNS-tropic AAVs under identical conditions is essential to establish a reliable basis for the rational selection of vectors in neuroscience research and gene therapy.

In this study, AAV9, rAAV2-retro, AAV-PHP.eB, and AAV-MacpnS1 were systematically compared following intravenous administration in neonatal C57BL/6 mice. The transduction efficiency and cellular tropism of all four AAVs in key CNS regions (the olfactory bulb, cortex, hippocampus, thalamus, cerebellum, medulla, and spinal cord), as well as in the DRG, heart, and liver, were quantitatively assessed. This research provides a comprehensive, “atlas-like” comparative framework that highlights the unique strengths and limitations of each capsid, ranging from global CNS coverage and neuronal subtype specificity to peripheral off-target effects. This offers practical guidance for selecting the most suitable AAV vector for neuroscience research and targeted gene therapy, particularly for neurodevelopmental and motor neuron disorders, where early, widespread or cell-specific intervention is critical.

## 2. Materials and Methods

### 2.1. Adeno-Associated Virus Vectors

All the recombinant AAV (rAAV) vectors used in this study—AAV9, rAAV2-retro, AAV-PHP.eB, and AAV-MacpnS1—were packaged and provided by BrainVTA Co., Ltd. (Wuhan, China). Each vector contained a genome expressing enhanced green fluorescent protein (eGFP) controlled by the cytomegalovirus (CMV) promoter. The viruses were produced in human embryonic kidney (HEK293T) cells using a standard triple-plasmid transfection protocol and were then purified using iodixanol gradient ultracentrifugation. The genomic titer (in vector genomes per milliliter, vg/mL) for each viral preparation was determined utilizing quantitative PCR (qPCR) [25]. For in vivo injections, all viral stocks were diluted in sterile 0.9% NaCl to a standard concentration. Each neonatal mouse received an absolute dose of 1 × 10^10^ vg in a 2 µL injection volume intravenously via the temporal vein.

### 2.2. Experimental Animals and Virus Injection

All animal experiments were conducted in accordance with the Fudan University Guidelines for the Care and Use of Laboratory Animals and were approved by the Animal Advisory Committee of Fudan University.

C57BL/6 mice (male and female) were obtained from SLAC Laboratory Animal Co., Ltd. (Shanghai, China) and were randomly assigned to experimental groups without sex-based stratification, as sex was not determined at the time of injection due to the difficulty in reliably identifying males from females at P2. The mice were housed under specific pathogen-free (SPF) conditions in the Fudan University Laboratory Animal Building. The temperature of the housing environment was maintained at 21–25 °C, and the relative humidity at 45–55%, with a 12-h light/dark cycle (lights on at 7:00). Food and water were provided ad libitum.

On postnatal day 2 (P2), pups were anesthetized. Anesthetization was achieved by placing the pups on ice for 30–60 s. For each animal, the target total viral dose was set to 1 × 10^10^ vg, and approximately 2 µL of undiluted viral stock was required according to viral titer. Direct loading of microliter-scale viral aliquots into 1 mL insulin syringes may introduce volumetric error. To minimize this error, injection mixtures were prepared on a sterile hydrophobic membrane: a 20 µL sterile saline droplet was dispensed first, followed by 2 µL concentrated viral stock delivered with a calibrated micropipette, and the solution was mixed gently. The full ~22 µL mixture was aspirated into a 1 mL insulin syringe fitted with a 29-gauge needle for injection. Any animal showing obvious liquid leakage, failed venipuncture, or immediate backflow during injection was excluded from subsequent analysis. All four capsid groups were processed by the same operator using identical dilution and loading workflows; any consistent volumetric error would therefore affect all groups equally, preserving the reliability of inter-group comparisons.

The entire viral mixture was administered to each pup via the temporal vein as follows: was administered to each pup via the temporal vein as follows: Under microscopic observation, the anesthetized pup was positioned with its right ear facing upward, and the head was stabilized. The needle was then carefully inserted into the temporal vein. Successful venipuncture was confirmed by visible blood reflux. At this point, the viral solution was injected slowly, and the needle was held in place for an additional 15 s to prevent backflow. To achieve hemostasis, gentle pressure was applied with a sterile cotton ball. The pups were then left to recover, after which they were gently rubbed with home-cage bedding to prevent maternal rejection and then returned to their cage.

### 2.3. Tissue Preparation and Immunofluorescence

Mice were deeply anesthetized with isoflurane and then transcardially perfused. Perfusion was carried out using ice-cold 1× PBS (Solarbio, Beijing, China), followed by fixation using freshly prepared, ice-cold 4% paraformaldehyde (PFA; Solarbio, Beijing, China) in 1× PBS. After perfusion, the brain, spinal cord (cervical segments C4–C7), dorsal root ganglia (DRGs; lumbar segments L1–L3), liver, and heart were excised from each mouse, then carefully dissected and post-fixed in 4% PFA at 4 °C for 48 h.

For immunofluorescence analysis, fixed tissues were embedded in 4.5% molten agarose and sliced into 35 μm thick sections using a vibratome (VT1200, Leica Biosystems, Nussloch, Germany). The brains were sliced sagittally, whereas the spinal cords, DRGs, hearts, and livers were sectioned coronally. The sections were quantified using a systematic random sampling approach, wherein every second section was selected for staining. Immunofluorescence staining was performed on free-floating sections using microwave-assisted heat-induced epitope retrieval (HIER) in preheated Tris-EDTA buffer (pH 9.0; (Solarbio, Beijing, China). Non-specific binding sites were blocked with 5% normal donkey serum (Solarbio, Beijing, China), and the sections were then incubated with primary antibodies for 16–20 h at 4 °C. The following primary antibodies were used: rabbit anti-Choline Acetyltransferase (ChAT; 1:1000; Abcam, Cambridge, UK, catalog No. ab178850), rabbit anti-NeuN (1:1000; Abcam, Cambridge, UK, catalog No. ab177487), and mouse anti-eGFP (1:1000; Santa Cruz Biotechnology, Dallas, TX, USA, catalog No. sc-9996). After incubation, the sections were thoroughly washed and then incubated with corresponding secondary antibodies for 2 h at room temperature. The secondary antibodies used were donkey anti-mouse Alexa Fluor 488 (1:500; Yeasen Biotechnology, Shanghai, China, catalog No. 34106ES60) and donkey anti-rabbit Alexa Fluor 647 (1:500; Yeasen Biotechnology, Shanghai, China, catalog No. 34213ES60). The nuclei were subsequently counterstained with DAPI (1:500; Sigma-Aldrich, St. Louis, MO, USA, catalog No. 10236276001), and the sections were mounted using an anti-fade medium (Beyotime, Shanghai, China, catalog No. P0126). For cell counting and co-localization analysis of eGFP, separate sections were immunostained with anti-eGFP primary antibody and Alexa Fluor 488 (a conjugated secondary antibody) to enhance cellular visualization.

### 2.4. Microscopy Imaging

Tissue sections were imaged using an Olympus FV3000 laser scanning confocal microscope (Olympus, Tokyo, Japan) fitted with a 20× objective. To examine the autofluorescence of native eGFP directly through eGFP fluorescence intensity quantification and eGFP^+^ cell counting, unstained sections were used. For normalization purposes, images of the identical tissue type and experimental batch were acquired using matched laser power, gain, offset, objective magnification, and Z-stack acquisition parameters. Standardized acquisition parameters were maintained across all imaging sessions to ensure consistency in fluorescence quantification. The following settings were applied to spot eGFP: brain sections were imaged at 488 nm excitation using 5% laser power and a gain of 470; the spinal cord ventral horn was excited at 488 nm using 5.5% laser power and a gain of 520; the spinal cord dorsal horn and DRGs were imaged at 488 nm excitation using 4.5% laser power and a gain of 420; the heart and liver sections were acquired at 488 nm excitation using 4.8% laser power and a gain of 430. ChAT immunoreactivity was captured at 647 nm excitation using 5% laser power and a gain of 550. Nuclei were counterstained with DAPI and imaged at 405 nm excitation using 3% laser power and a gain of 560. Images were taken using Z-stack to ensure comprehensive cellular coverage. The brain, spinal cord, heart, and liver tissues were imaged as 17 optical sections with a Z-interval of 2-μm. Whole-mounted DRG tissues (the L2 segment) were imaged as 12 optical sections with a Z-interval of 8-μm. High-resolution Z-stacks were obtained for cardiac and hepatic tissues with 0.3-μm Z-steps and for neural tissues with 0.5-μm Z-steps. To minimize variability, identical acquisition settings within the same imaging session were maintained for each tissue and region across all experimental groups. The order of selection of slides for imaging was randomized, and the investigators were blinded to the experimental groups during image acquisition and the initial analysis. The fluorescence intensity quantification and the counting of eGFP-positive cells were carried out using ImageJ software (v1.53, NIH); background subtraction was applied uniformly to all regions of interest.

### 2.5. Quantitative and Statistical Analysis

Images of tissue sections in the experimental groups were quantified blindly using ImageJ (v1.53, NIH). For fluorescence intensity measurements, regions of interest (ROIs) consisting of specific anatomical structures (e.g., the olfactory bulb, cortex, hippocampus, thalamus, cerebellum, medulla, spinal cord horns, DRG, heart, and liver) were outlined manually. The corrected mean fluorescence intensity (MFI) for eGFP was calculated as: MFI = MFI_ROI_ − MFI_Background_. ROIs were manually outlined following anatomical landmarks to standardize region selection across samples, and background signals from adjacent low-fluorescence non-target areas within the same section were quantified.

eGFP-positive (eGFP^+^) cells were defined as those with a distinct cellular morphology and a fluorescence intensity greater than three standard deviations above the background level. For each animal, serial sagittal brain sections (35 μm) were collected from a mediolateral range of 0.24–1.44 mm lateral to the midline, covering all target brain regions. To ensure comparability and systematic spatial coverage between animals, confocal imaging and subsequent cell counting were performed for every other section within this range. eGFP^+^ cells in each ROI were counted manually, and the data for each animal were expressed as the average number of eGFP^+^ cells per section per region. To determine cell type-specific transduction, the proportions of transduced neurons and cholinergic motor neurons were calculated as the percentages of eGFP^+^ cells that were co-labeled with NeuN (eGFP^+^NeuN^+^/eGFP^+^ × 100%) and ChAT (eGFP^+^ChAT^+^/eGFP^+^ × 100%), respectively.

At least four biological replicates (individual mice) were carried out per group, and the data from their analyses are expressed as mean ± standard error of the mean (SEM). The differences between the groups were analyzed using one-way ANOVA, followed by Tukey’s multiple-comparisons test. Statistical analysis and graphing were conducted using GraphPad Prism (v10.3). Significance was set at * *p* < 0.05, ** *p* < 0.01, and *** *p* < 0.001.

## 3. Results

### 3.1. The Distribution of Transduction and the Expression Intensity of Four AAV Capsids in Selected CNS Regions

In order to systematically evaluate the transduction profiles of the different AAV capsids in the CNS, AAV9, rAAV2-retro, AAV-PHP.eB, and AAV-MacpnS1, all of which expressed eGFP under the control of the CMV promoter, were separately injected into the temporal vein of neonatal mice (P2). The mice were sacrificed on P30, after which analyses were performed. Accordingly, 1 × 10^10^ vg was selected as the dose per pup based on findings from prior studies on neonatal intravenous AAV that had demonstrated robust CNS transduction without acute adverse effects [12]. For a P2 pup (~2.0 g body weight), this corresponds to approximately 5.0 × 10^12^ vg/kg.

**Figure 1:**
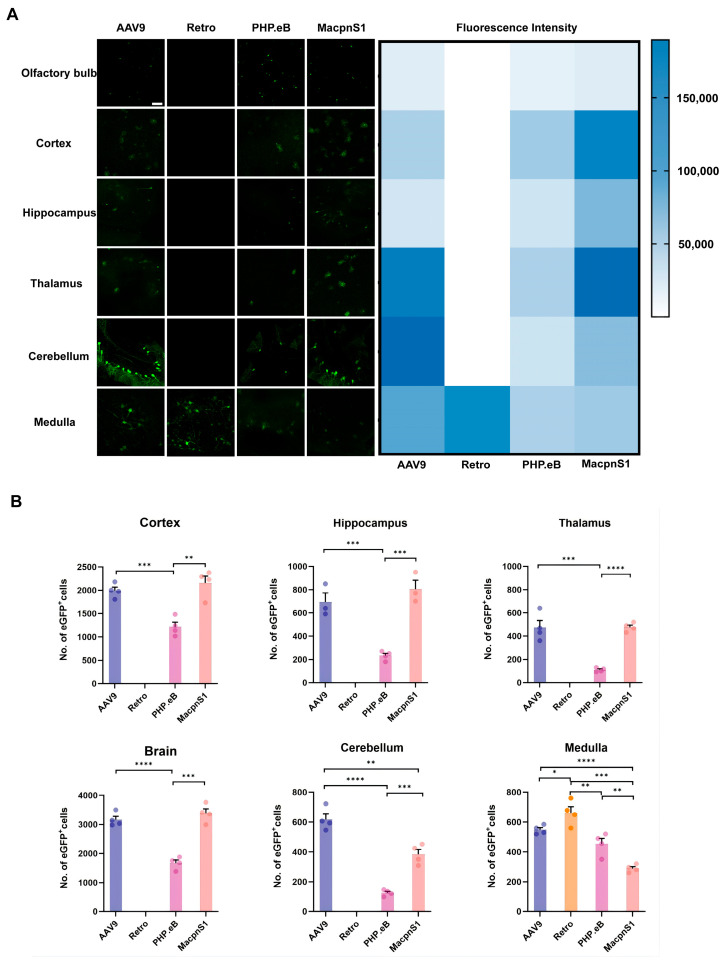
**Different transduction profiles of four AAV capsids in the mouse brain following intravenous administration.** (**A**) Representative immunofluorescence images showing eGFP expression in the olfactory bulb (OB), cortex (Ctx), hippocampus (Hip), thalamus (Tha), cerebellum (Cb), and medulla (Med) of mice 28 days after receiving an IV injection of eGFP-expressing AAV9, rAAV2-retro, AAV-PHP.eB, or AAV-MacpnS1 through the temporal vein as neonates (1 × 10^10^ vg per mouse). Scale bar = 50 µm. The Heatmap (on the right) quantifies the mean eGFP fluorescence intensity (arbitrary units/pixel) across brain regions (*n* = 5 mice per group). (**B**) Quantification of the number of eGFP^+^ cells transduced by each capsid in the specified brain regions. Data represent the mean ± SEM (*n* = 4 mice per group, individual dots represent the value of each individual mouse). Statistical significance was determined using one-way ANOVA, followed by Tukey’s multiple comparisons (* *p* < 0.05; ** *p* < 0.01; *** *p* < 0.001; **** *p* < 0.0001).

As revealed through immunofluorescence, the transduction patterns across the capsids were distinct. AAV9, AAV-PHP.eB, and AAV-MacpnS1 were broadly transduced in multiple CNS regions, including the olfactory bulb, cortex, hippocampus, thalamus, medulla oblongata, and cerebellum (Figure 1A), albeit with varying expression profiles. Per the quantitative assessment of the signal intensity (mean intensity per pixel) of eGFP in these regions, the signals of AAV9 in the thalamus and cerebellum were highly intense. AAV-MacpnS1 was more efficiently transduced in the thalamus and cortex than AAV9. While the transduction of AAV-PHP.eB was also widespread, its fluorescence intensity was relatively weak across all examined regions. In stark contrast, the expression of rAAV2-retro was markedly region-specific, with detectable eGFP signals confined exclusively to the medulla (Figure 1A). These findings were further corroborated by the outcome of the statistical analysis of the number of eGFP-positive cells. They showed that AAV9 and AAV-MacpnS1 produced the highest number of transduced cells across multiple brain regions, including in the cerebellum (Figure 1B). The overall ranking for the number of infected cells throughout the brain and cerebellum was as follows: AAV9 ≈ AAV-MacpnS1 > AAV-PHP.eB. Despite its restricted expression pattern, rAAV2-retro’s transduction in the medulla was the highest (675.25 ± 35.36 eGFP^+^ cells) (Figure 1B).

**Figure 2:**
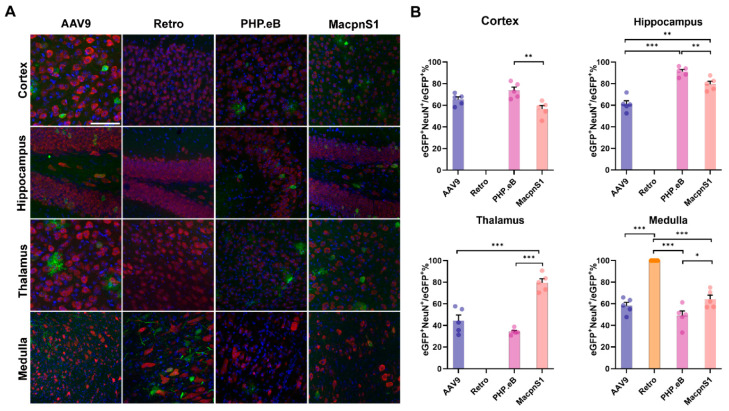
**Neuronal tropism of the AAV capsids in the mouse brain following intravenous administration.** (**A**) Representative confocal micrographs of double-immunofluorescence staining in brain sections. eGFP^+^ cells (green) co-localize with the neuronal marker NeuN (red). Nuclei were counterstained with DAPI (blue). Scale bar: 50 μm. (**B**) Quantification of neuronal transduction efficiency expressed as the percentage of eGFP^+^ cells co-labeled with NeuN^+^ in the cortex, hippocampus, thalamus, and medulla. Data represent the mean ± SEM (*n* = 5 per group, individual dots represent the value of each individual mouse). Statistical significance was determined using one-way ANOVA, followed by Tukey’s multiple comparisons (* *p* < 0.05; ** *p* < 0.01; *** *p* < 0.001).

To determine the proportion of transduced cells that were neurons, neuronal tropism was assessed after co-staining sections with NeuN (a neuronal marker) and eGFP antibodies. The ratio of eGFP^+^NeuN^+^ cells to total eGFP^+^ cells (eGFP^+^NeuN^+^/eGFP^+^) was then calculated to determine neuronal transduction efficiency (Figure 2A,B). In the cortical region, the neuronal transduction ratios for AAV9, AAV-PHP.eB, and AAV-MacpnS1 were similar (68.38 ± 3.45%, 68.37 ± 7.57%, and 60.73 ± 4.09%, respectively). In the hippocampus, however, the neuronal transduction ratio for AAV-PHP.eB was significantly higher (90.86 ± 3.98%) than those for both AAV9 (62.47 ± 7.53%) and AAV-MacpnS1 (79.9 ± 4.01%). AAV-MacpnS1’s ratio in the hippocampus was also significantly higher than that for AAV9, and its neuronal tropism in the thalamus was unique, with a neuronal transduction ratio (79.54 ± 6.39%) approximately twice that of AAV9 (43.44 ± 10.37%) or AAV-PHP.eB (33.55 ± 2.48%). Notably, for rAAV2-retro, whose expression was only observed in the medulla after administration through IV injection, 100% of the eGFP^+^ signals detected co-localized with NeuN^+^, suggesting that the transduction of neurons in this region was exclusive.

### 3.2. Transduction of the AAV Capsids in the Spinal Cord

**Figure 3:**
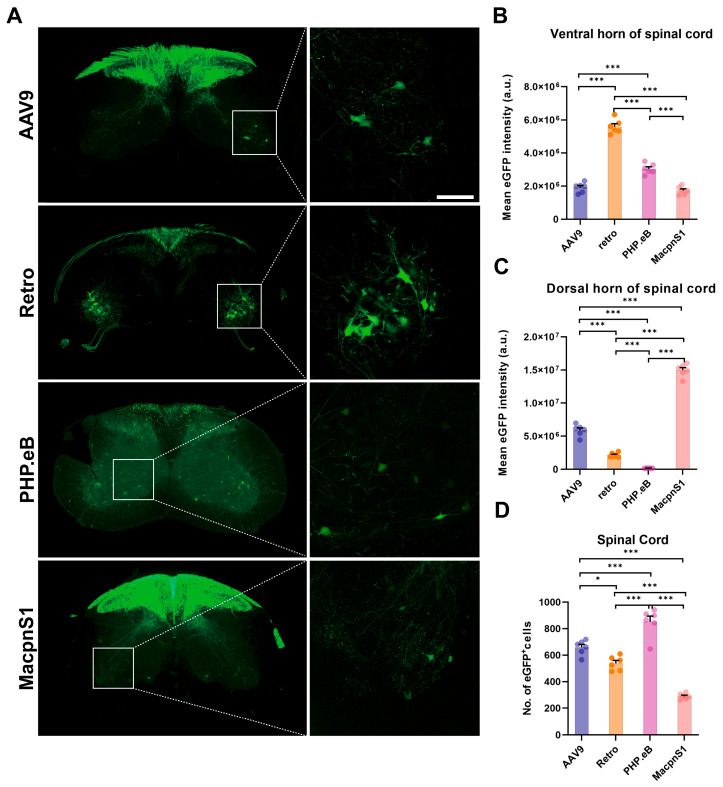
**Transduction efficiency of the AAV capsids in the mouse spinal cord following intravenous administration.** (**A**) Representative fluorescence images of cervical spinal cord sections from mice injected intravenously with AAV9, rAAV2-retro, AAV-PHP.eB, or AAV-MacpnS1 on postnatal day 2 (P2), analyzed 28 days post-injection (1 × 10^10^ vg per mouse; *n* = 6 per group, individual dots represent the value of each individual mouse.). The images show the expression of eGFP as driven by the CMV promoter. The boxed regions indicate the ventral horn, with corresponding magnified views shown in the right panels. Scale bars: 200 µm (overview); 50 µm (magnification). Scale bars: 200 µm (overview); 50 µm (magnification). (**B**) Quantification of the fluorescence intensity of eGFP (mean intensity per pixel) in the ventral horn. (**C**) Quantification of the fluorescence intensity of eGFP (mean intensity per pixel) in the dorsal horn. (**D**) Quantification of eGFP-positive cells in the spinal cord. Data are presented as the mean ± SEM. Statistical significance was determined using one-way ANOVA, followed by Tukey’s multiple comparisons (* *p* < 0.05; *** *p* < 0.001).

In the assessment for transduction efficiency, all four capsids showed notable transduction throughout the spinal cord (Figure 3A). According to semi-quantitative fluorescence analysis, rAAV2-retro and AAV-PHP.eB were responsible for the highest eGFP expression intensities in the ventral horn (Figure 3B). Their expression levels in the dorsal horn were, however, comparatively low. Notably, the mean eGFP fluorescence intensity in the dorsal horn in the AAV-MacpnS1 group was significantly higher than that for each of the other three capsids (*p* < 0.001) (Figure 3C). Quantifying the number of eGFP-positive cells in the ventral horn (Figure 3D) revealed that the number of AAV-PHP.eB cells transduced in this region was considerably higher than observed for the other capsids (AAV-PHP.eB: 847.4 ± 125.56; AAV9: 662.83 ± 20.57; rAAV2-retro: 535.33 ± 23.38; AAV-MacpnS1: 291 ± 8.92).

**Figure 4:**
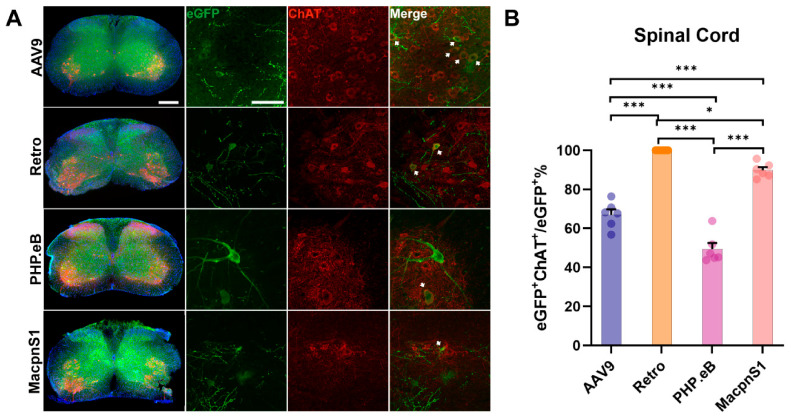
**Comparison of the transduction efficiency of the AAV capsids in the cholinergic motor neurons of the mouse spinal cord.** (**A**) Representative immunofluorescence images of cervical spinal cord sections showing eGFP expression (green) in ChAT^+^ motor neurons (red). The arrowheads indicate eGFP^+^ cells co-labeled with ChAT^+^ cholinergic motor neurons. Nuclei were counterstained with DAPI (blue). Scale bars: 100 µm (main panels); 50 µm (insets). (**B**) Percentage of eGFP^+^ neurons co-localized with ChAT^+^ motor neurons (ChAT^+^/eGFP^+^). Data are presented as the mean ± SEM (*n* = 6 mice per group, individual dots represent the value of each individual mouse). Statistical significance was determined using one-way ANOVA, followed by Tukey’s multiple comparisons (* *p* < 0.05; *** *p* < 0.001).

To specifically assess the tropism of the capsids for motor neurons, they were co-stained for their affinity to eGFP and choline acetyltransferase (ChAT), a specific marker for cholinergic motor neurons (Figure 4A). Quantifying the percentage of eGFP^+^ cells that were also ChAT^+^ (eGFP^+^ChAT^+^/eGFP^+^) unveiled distinct capsid-specific profiles (Figure 4B). rAAV2-retro demonstrated exclusive tropism for cholinergic motor neurons, with 100% of its eGFP^+^ cells co-localizing with ChAT^+^. AAV-MacpnS1 likewise showed high efficiency, with 87.3 ± 3.3% of the eGFP^+^ cells identified as motor neurons. For AAV9, 70.7 ± 4.8% of the transduced cells were ChAT-positive. However, while the highest total number of cells transduced in the ventral horn was AAV-PHP.eB (Figure 3D), AAV-PHP.eB displayed the lowest preference for cholinergic motor neurons, with only 53.8 ± 7.5% of its eGFP^+^ cells co-localizing with ChAT^+^. We note here that the eGFP signal intensity in Figure 4 appears lower than that in Figure 3. This is due to requiring heat-induced epitope retrieval (HIER) for ChAT immunostaining, which partially quenches the fluorescence of endogenous eGFP. After HIER, the signal was recovered using anti-eGFP immunostaining.

### 3.3. Transduction in the PNS After Intravenous AAV Administration

**Figure 5:**
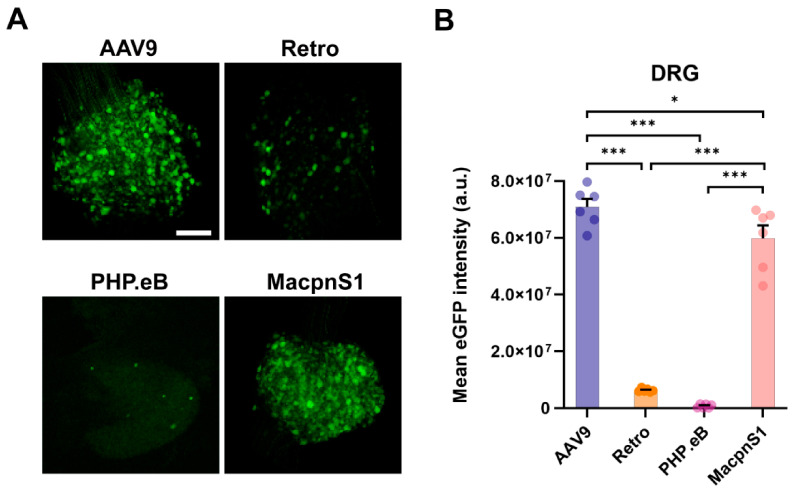
**Comparative transduction efficiency of the AAV capsids in the dorsal root ganglia.** (**A**) Representative immunofluorescence images of lumbar DRG sections transduced by AAV capsids. The expression of eGFP (green) marks the transduced sensory neurons. Nuclei were counterstained with DAPI (blue). Scale bar: 100 µm. (**B**) Quantification of the mean fluorescence intensity of eGFP (per pixel) in DRG neurons. Data are presented as the mean ± SEM (*n* = 6 mice per group, individual dots represent the value of each individual mouse). Statistical significance was determined using one-way ANOVA, followed by Tukey’s multiple comparisons (* *p* < 0.05; *** *p* < 0.001).

The potential of AAV transduction in peripheral neuronal tissues after systemic delivery was next evaluated by examining the efficiency of transduction in the DRG (Figure 5). The quantitative analysis of the fluorescence intensity of eGFP revealed varying outcomes for the capsids. The transduction of both AAV9 and AAV-MacpnS1 in DRGs was highly efficient. In contrast, rAAV2-retro and AAV-PHP.eB exhibited only minimal eGFP expression levels, resulting in fluorescence intensities of eGFP approximately 4- to 6-fold lower than those of AAV9 and AAV-MacpnS1 (Figure 5B). Notably, AAV-MacpnS1 had the highest eGFP expression levels of all four capsids in the DRGs displaying transduction, suggesting that it had superior tropism for sensory neurons in this peripheral ganglion compared to the other capsids.

### 3.4. Transduction in Cardiac and Hepatic Tissues After Systemic AAV Administration

Because the heart and liver are major peripheral targets for many AAV capsids, their transduction profiles are highly relevant when it comes to assessing targeting specificity and potential off-target effects. The transduction efficiency of each of the four AAV capsids was therefore evaluated in these organs.

**Figure 6:**
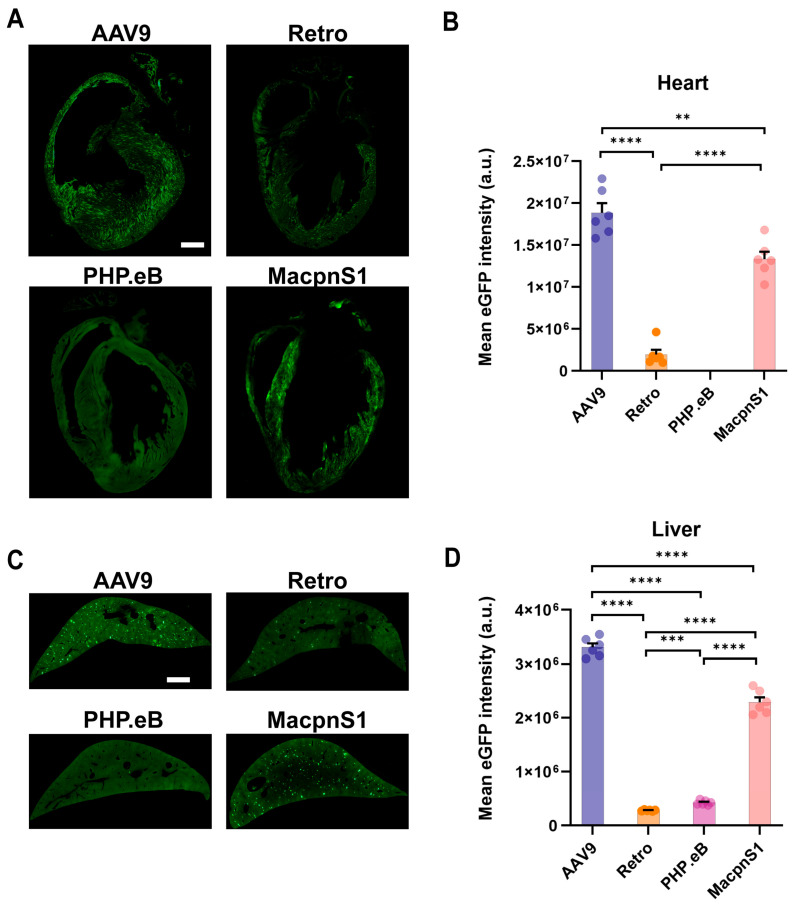
**Comparative transduction efficiency of the AAV capsids in cardiac and hepatic tissues.** (**A**) Representative immunofluorescence images of heart sections showing the expression of eGFP (green) 28 days post-intravenous injection. Scale bar: 1000 µm. (**B**) Quantification of the mean fluorescence intensity of eGFP (per pixel) in cardiac tissues. (**C**) Representative immunofluorescence images of liver sections showing the expression of eGFP (green) 28 days post-intravenous injection. Scale bar: 200 µm. (**D**) Quantification of the mean fluorescence intensity of eGFP (per pixel) in hepatic tissues. Data represent the mean ± SEM (*n* = 6 mice per group, individual dots represent the value of each individual mouse). Statistical significance was determined using one-way ANOVA, followed by Tukey’s multiple comparisons (** *p* < 0.01; *** *p* < 0.001; **** *p* < 0.0001).

Imaging and subsequent quantification of fluorescence intensity revealed distinct transduction profiles in the heart tissues (Figure 6A,B). Both AAV9 and AAV-MacpnS1 demonstrated high cardiac transduction efficiency. In contrast, rAAV2-retro and AAV-PHP.eB were relatively weakly transduced in the heart. A similar pattern was observed in the liver (Figure 6C,D), where the highest transduction levels were again registered for AAV9 and AAV-MacpnS1, followed by rAAV2-retro and then AAV-PHP.eB. This consistent trend highlights the similarity between AAV9 and AAV-MacpnS1, which influence peripheral tropism, resulting in robust transgene expression in both the nervous system and key peripheral organs.

### 3.5. Biochemical Indicators of Hepatic Stress Assessment After Systemic AAV Administration

Given the high levels of transduction observed in the liver for several capsids, particularly AAV9 and AAV-MacpnS1, the potential biochemical indicators of hepatic stress associated with systemic vector administration were next evaluated. The levels of alanine aminotransferase (ALT), aspartate aminotransferase (AST), alkaline phosphatase isozyme 2c (ALP-2c), and total protein (TP) in the serum were measured 28 days post-injection.

**Figure 7:**
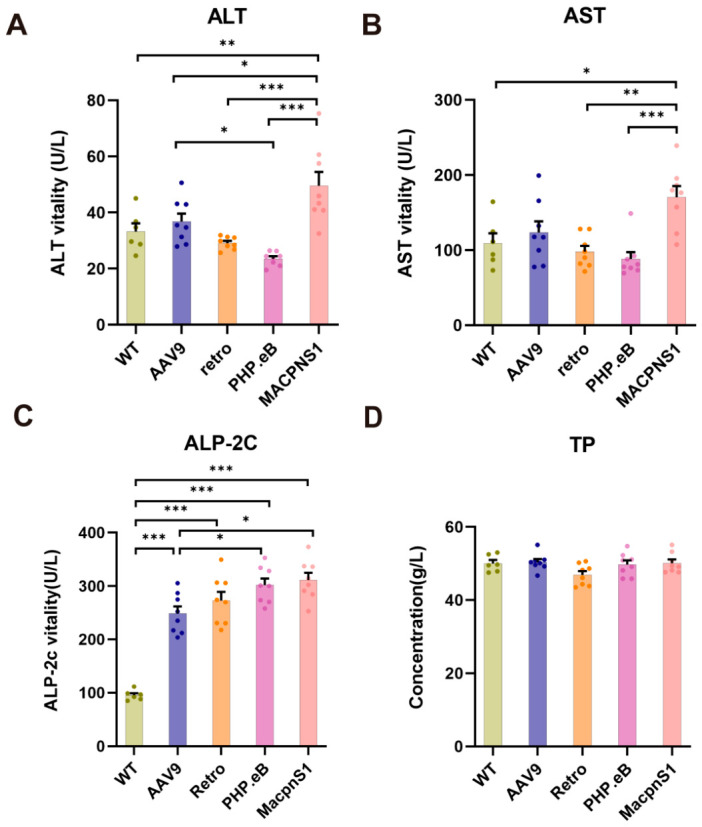
**Assessment of biochemical indicators of hepatic stress following the systemic administration of the AAV capsids.** (**A**) Serum alanine aminotransferase (ALT) activity. (**B**) Serum aspartate aminotransferase (AST) activity. (**C**) Serum alkaline phosphatase isoform 2c (ALP-2c) activity. (**D**) Total protein (TP) concentration. Data represent the mean ± SEM (*n* = 8 mice per group, individual dots represent the value of each individual mouse) measured 28 days post-intravenous injection. Wild-type (WT) mice without injections served as controls. Statistical significance was determined using one-way ANOVA, followed by Tukey’s multiple comparisons (* *p* < 0.05; ** *p* < 0.01; *** *p* < 0.001).

Compared to wild-type (WT) controls, serum ALT and AST levels remained largely stable across three of the AAV-injected groups. Only the AAV-MacpnS1 group registered significant increases in both (Figure 7A,B). In contrast, ALP-2c levels were elevated in all four groups compared to WT controls. The most substantial increase was registered in the AAV-MacpnS1 group (Figure 7C). Nevertheless, there were no significant changes in TP levels in all four experimental groups (Figure 7D). This suggests the preservation of synthetic function. An increase in serum transaminase alone does not definitively establish hepatotoxicity, as enzyme leakage can occur under cellular stress without structural damage. Still, these data suggest that all four capsids, particularly AAV-MacpnS1, possibly impacted the liver, at least to a degree, although there appeared to be no effect on synthetic liver function.

## 4. Discussion

The comprehensive evaluation of four AAV capsids—AAV9, rAAV2-retro, AAV-PHP.eB, and AAV-MacpnS1—after they were administered intravenously to neonatal mice revealed their distinct transduction profiles across the CNS and PNS. The transduction of AAV9 and AAV-MacpnS1 in the CNS was widespread, with the latter exhibiting superior neuronal tropism in the thalamus. By contrast, the tropism of rAAV2-retro was highly specific to the medulla and spinal motor neurons. While AAV-PHP.eB was broadly transduced in the spinal cord, it was rather lowly transduced in cholinergic motor neurons. Additionally, AAV9- and AAV-MacpnS1-driven expression of transgenes in the DRG and peripheral organs was robust. However, AAV-MacpnS1 was associated with notable biochemical indicators of hepatic stress. Nevertheless, these results provide a framework for identifying the unique advantages and limitations of each capsid for gene delivery to the mammalian nervous system.

Our findings align with and extend previously reported transduction profiles of these capsids under different experimental conditions, revealing critical age- and route-dependent differences. For AAV9, the widespread neuronal transduction we observed in neonates is consistent with studies showing that neonatal intravenous AAV9 preferentially targets neurons, whereas adult administration shifts tropism toward astrocytes [12]. This age-dependent switch highlights that our data represent the “early-life” profile relevant to neurodevelopmental disorders. Of note, intramuscular injection of AAV9 has also been shown to achieve widespread spinal cord transduction in both neonatal and adult mice, albeit with different efficiencies and ipsilateral bias in neonates [26]. For AAV-PHP.eB, its broad CNS distribution in neonatal C57BL/6J mice mirrors reports in adult C57BL/6J mice [17]. However, its strain dependence via the Ly6a receptor [27] limits generalizability. Notably, despite transducing the highest number of spinal cord cells, it exhibited the lowest specificity for cholinergic motor neurons (~54%), suggesting broad but non-selective transduction. For rAAV2-retro, we previously reported that intramuscular injection achieves efficient retrograde transduction of lower motor neurons in the spinal ventral horn and brainstem motor nuclei [16]. Here, we show that intravenous delivery produces a highly similar profile—transgene expression also localizes to the medulla and spinal ventral horn with 100% neuronal specificity—indicating that the capsid’s targeting properties are largely preserved across administration routes. For AAV-MacpnS1, a previous study demonstrated efficient PNS transduction but low CNS transduction in adult mice [19], whereas our neonatal data reveal widespread CNS transduction across multiple brain regions, highlighting the critical role of an immature BBB. Importantly, while that study reported lower liver transduction in adult mice compared to AAV9, we observed robust hepatic transduction and significant serum liver enzyme elevations in neonates, suggesting age-dependent differences in hepatic safety. Collectively, these comparisons demonstrate that transduction efficiency, tropism, and safety are profoundly influenced by developmental stage and administration route, underscoring the importance of matching vector selection to the specific experimental or therapeutic context.

Systematically comparing AAV capsid-related transduction efficiency and tropism in the neonatal murine nervous system is particularly valuable for modeling neurodevelopmental diseases and therapeutic intervention. In neonates, the BBB is more permeable, and neurodevelopment is ongoing, which presents a unique biological window. This immaturity of the BBB is likely the reason for the increased CNS transduction observed here compared to profiles typically reported in adult mice [28]. This study therefore provides a critical, developmentally contextualized “vector atlas” for targeting the nervous system during its formative stages. This is especially important for early-onset genetic disorders, as intervention before the onset of irreversible pathology is crucial. These data can serve to guide the selection of the most suitable vector for achieving the desired spatial and cellular transduction pattern at the earliest time point, which is potentially the most therapeutically impactful period.

Key trade-offs associated with AAV delivery routes are also highlighted here. Unlike invasive neurosurgical methods, such as ICV, intrathecal (IT), spinal subpial, and intraparenchymal injections, IV delivery is minimally invasive. Its widespread transduction in the CNS of neonates in particular is crucial for treating disorders with diffuse pathology. However, unlike IM injection, which is highly efficient for retrograde targeting of specific motor neuron pools, IV administration provides broader, “top-down” access to various CNS regions (e.g., the cortex and thalamus) and peripheral ganglia, as demonstrated by the actions of AAV9 and AAV-MacpnS1. The main issue with IV delivery is balancing transduction in the CNS with off-target effects, particularly biochemical indicators of hepatic stress. This investigation reveals a critical capsid-dependent spectrum of off-target hepatic biochemical alterations that range from prominent biochemical indicators of hepatic stress induced by AAV-MacpnS1 to the minimal perturbations observed with rAAV2-retro. Accordingly, the most suitable strategy is context-dependent: IM delivery offers precision for motor neuron circuits, while IV delivery provides breadth for global CNS targets, necessitating a capsid with an acceptable safety profile.

AAV9 has been shown to play a robust and versatile role in the widespread delivery of genes across the CNS and peripheral tissues, and that role was here validated. Its capability to mediate substantial transduction in numerous brain regions, including the cortex, hippocampus, and cerebellum, as well as in the spinal cord, was here reinforced. Notably, its efficient transduction in the thalamus and DRGs, similar to that of MacpnS1, highlights its utility for sensory neuroscience. However, its broad tropism is a double-edged sword. AAV9 also mediated high levels of off-target expression in the liver and heart. This correlated with moderate biochemical indicators of hepatic stress, which were less severe than those caused by AAV-MacpnS1. This balance between widespread CNS efficacy and manageable peripheral toxicity establishes AAV9 as a “workhorse” capsid, making it particularly suitable for therapeutic strategies that require global transgene distribution within the nervous system, such as those targeting lysosomal storage disorders [29], or for delivering neuroprotective factors to broad neuronal populations. Its safety profile, which is well-documented in clinical trials, also supports its use in scenarios where balancing comprehensive CNS delivery with an acceptable systemic safety window is critical.

The transduction profile of AAV-PHP.eB was strikingly unique and somewhat paradoxical. It enabled broad distribution across multiple brain regions. Most particularly, the total number of AAV-PHP.eB cells transduced in the spinal cord ventral horn was higher than the number for each of the other capsids tested. However, this impressive quantitative efficiency was accompanied by a critical qualitative limitation: strikingly low specificity for motor neurons. Per the co-localization analysis with ChAT, fewer than 54% of the eGFP^+^ cells in the ventral horn were cholinergic motor neurons—the lowest proportion observed among the capsids tested. This suggests that AAV-PHP.eB drives the transduction of a sizable proportion of non-ChAT^+^ cells in this region, possibly including interneurons and glial cells. This discrepancy between high total transduction and low cellular specificity limits its application. Nevertheless, AAV-PHP.eB is a powerful vector for strategies that require widespread gene delivery across the CNS parenchyma, independent of neuron-specific expression. It is ideally suited to manipulating the local spinal cord circuitry, modulating the glial environment in conditions such as neuroinflammation or pain, and delivering secreted factors that can act in a paracrine manner [30]. Another critical shortcoming of AAV-PHP.eB is its strain-dependent transduction efficiency. The robust CNS transduction observed in this study, as well as in previous reports, relies on the expression of the Ly6a receptor, which is highly expressed in C57BL/6 mice but is largely absent or functionally variant in other mouse strains such as BALB/c [27]. Therefore, these findings with AAV-PHP.eB are specific to the C57BL/6 background and may not generalize to other strains. Importantly, Ly6a and Ly6c1 are absent in primates, which represents a translational limitation for AAV-PHP.eB-based gene therapy. Hence, while AAV-PHP.eB represents a pinnacle of engineered efficiency in murine models, it is best used for preclinical proof-of-concept studies targeting non-cell-autonomous mechanisms within the CNS.

We previously established rAAV2-retro as a highly efficient vector for lower motor neuron transduction following intramuscular injection [16]. Building on that finding, this study consolidates its unique value through a systematic comparative analysis. After IV delivery, rAAV2-retro once again was exceptionally specific in action, with transgene expression predominantly confined to the medulla and spinal motor neurons, as evidenced by its near-complete co-localization with ChAT. This consistent performance across administration routes contrasts starkly with the broader tropism of AAV9, AAV-PHP.eB, and AAV-MacpnS1 and underscores rAAV2-retro’s inherent and robust retrograde trafficking capability. Critically, this precise neural targeting is complemented by a comparatively favorable systemic safety profile. In the assessment of biochemical indicators of hepatic stress, no significant changes in ALT, AST, or TP levels were recorded in the presence of rAAV2-retro compared to wild-type controls, despite an elevation in ALP-2c. This combination of high target neuron efficiency and a favorable systemic safety profile reaffirms its value for both basic research and clinical translation. Its tropism may be confined, but the potency of that tropism makes it an indispensable tool for dissecting motor circuits, enabling precise genetic interrogation in models of hereditary spastic paraplegia or spinal muscular atrophy. When integrated with optogenetic actuators (e.g., ChR2) or chemogenetic systems (e.g., DREADDs), it serves as a powerful platform for preclinical validation, enabling the real-time functional mapping of motor ensembles and allowing the efficacy of therapeutic interventions to be quantitatively assessed. Furthermore, rAAV2-retro’s abilities position it as a premier platform for in vivo gene therapy. Transduction in the pan-spinal motor neuron via minimally invasive IM or IV injection has been transformative in the treatment of motor neuron diseases (MNDs). This vector enables therapeutic genes to be delivered directly to the primary cells affected in MNDs for neuroprotection, gene silencing, and regenerative factors. The confirmed efficacy of these minimally invasive delivery routes and rAAV2-retro’s demonstrated safety greatly enhance clinical feasibility. However, efforts must be undertaken to refine rAAV2-retro’s tropism and mitigate immune responses in large-animal models if this precise targeting capability is to be translated into transformative gene therapies for motor neuron disorders.

AAV-MacpnS1 was here identified as a highly distinctive and potent capsid with a unique dual-compartment targeting profile. While it rivals AAV9 in its ability to mediate widespread transduction throughout the CNS, it displays significantly superior neuronal tropism in the thalamus, infecting nearly twice the proportion of neurons compared to AAV9. This makes AAV-MacpnS1 an exceptional tool for probing thalamic circuitry in sensory processing and integration. Furthermore, AAV-MacpnS1 is remarkably efficient in the PNS, driving robust transduction in DRG neurons and the spinal dorsal horn to a degree comparable with, or even exceeding, that achieved by AAV9. This combination of efficiently targeting both central and peripheral sensory neurons is rare and highly valuable. However, this broad and efficient tropism comes at the cost of considerable biochemical indicators of hepatic stress. In its presence, ALT, AST, and ALP-2c levels increased significantly, indicating acute liver injury. This poses a major obstacle to AAV-MacpnS1’s clinical application ability. Consequently, AAV-MacpnS1 should be regarded primarily as a premier vector for robustly targeting central and sensory pathways in preclinical animal models, particularly in research on chronic pain, itch, and thalamocortical communication. The potential for its use in the treatment of chronic pain is noteworthy. Administering AAV-MacpnS1 intravenously can target both DRG sensory neurons and their synapses in the spinal dorsal horn simultaneously and deliver key molecules that inhibit pain signaling and thus intervene in the transmission of pain signals. Still, AAV-MacpnS1-related biochemical indicators of hepatic stress must be carefully considered moving forward. To mitigate toxicity, strategies such as capsid engineering (e.g., designing mutants with low hepatic targeting or immune evasion sites) and the use of tissue-specific promoters (e.g., cTnT or Syn1) should be explored, as they could limit transgene leakage [31]. Furthermore, combining AAV-MacpnS1 with immunomodulatory interventions (e.g., glucocorticoids or miRNA regulatory elements to degrade non-target tissue mRNA) could optimize vector design and enhance therapeutic safety [32,33].

The biochemical analysis for this research was performed for a single time point only (day 28 post-injection), a time point at which tissue was harvested. This is a limitation for the study, as it means that the early dynamics of ALT/AST elevation were not assessed. Future studies should incorporate early time points (e.g., day 7 and day 14) to fully characterize the temporal profile of hepatic responses. Additionally, serum liver biochemistry alone cannot reflect complete hepatic alterations caused by AAV vectors, as serum enzyme markers alone do not fully capture vector-related hepatic effects. Future studies should incorporate qPCR quantification of AAV genome copies in liver tissues, as well as histopathological examinations to comprehensively assess the safety profile of these capsids. Furthermore, a limitation of our safety assessment is the focus on serum liver enzyme markers without evaluation of broader immune responses, including cytokine profiles, anti-AAV antibody production, or complement activation. Future studies incorporating immunological endpoints will be necessary to fully characterize the safety profile of these capsids following systemic delivery in neonates.

Four AAV capsids were assessed in this study and revealed to have significant complementary transduction characteristics in both the central and peripheral nervous systems. This provides important theoretical support for the combined application of multiple capsids in disease treatment. For instance, combining AAV-MacpnS1 with rAAV2-retro in chronic pain models enables dual intervention, as it would address both pain signal transmission and deficits in motor control often associated with chronic pain conditions. Similarly, combining rAAV2-retro with AAV-PHP.eB in diseases like ALS could reduce the risk of hepatic toxicity by lowering the single dosage and targeting a wider range of pathological targets. Furthermore, incorporating optimization strategies, such as tissue-specific promoters or capsid engineering, could enhance the safety and precision of combination therapies even further. The feasibility of combining treatment strategies has already been trialed in fields such as deafness and hemophilia [34,35,36]. However, this strategy of combining therapies presents potential challenges, including the accumulation of immunogenicity from different capsids, determining the optimal administration time, and competitive receptor binding between vectors. It is important to note that the enhanced CNS transduction observed in this neonatal model is partly due to the immature BBB at P2, which is more permissive to AAV passage than the intact BBB in adult humans. Therefore, our findings should not be directly extrapolated to adult CNS applications. Instead, these data are most relevant to early-intervention strategies for neurodevelopmental disorders, where the neonatal period offers a unique therapeutic window.

## Data Availability

All data generated or analyzed during this study are included in this published article. Raw data are available from the corresponding author upon reasonable request.
